# Impact of trypanosomiasis on male camel infertility

**DOI:** 10.3389/fvets.2024.1506532

**Published:** 2025-01-16

**Authors:** Sara Salah Abdel-Hakeem, Gaber Megahed, Ahmed M. Al-Hakami, Mohammed E. M. Tolba, Yasser F. M. Karar

**Affiliations:** ^1^Parasitology Laboratory, Department of Zoology and Entomology, Faculty of Science, Assiut University, Asyut, Egypt; ^2^Department of Veterinary Theriogenology, Faculty of Veterinary Medicine, Assiut University, Asyut, Egypt; ^3^Department of Clinical Microbiology and Parasitology, College of Medicine, King Khalid University, Abha, Saudi Arabia; ^4^Zoology and Entomology Department, Faculty of Science, New Valley University, El-Kharga, Egypt

**Keywords:** *Camelus dromedarius*, fertility, HSP70, proteomic, testicular lesion, *Trypanosoma evansi*

## Abstract

**Introduction:**

Blood parasitism is a significant clinical disease that silently undermines the livestock industry, particularly affecting camels. This study aimed to assess the prevalence of *Trypanosoma evansi* in Arabian camels (*Camelus dromedarius*) and its impact on infertility by examining serum protein fractions, lipids, reproductive indices, and the expression of heat shock protein (HSP70) during breeding season.

**Methods:**

A total of 107 male post-pubertal camels, aged between 5 and 10 years, were collected randomly from slaughtering house in Assiut Governorate, Egypt.

**Results:**

Microscopic and serological examinations revealed that 23.4% (25/107) of the camels were infected with *T. evansi*. Infected camels exhibited a highly significant increase in total serum protein. The assessment of dyslipidemia, measure as binary variables for lipid profiles (cholesterol, triglycerides, HDL, and LDL), indicated a nonsignificant increase in risk of dyslipidemia in infected camels compared to healthy camels. Proteomic analysis identified four major protein fractions in the infected camels compared to healthy camels with molecular weights of 181.72, 87.59, 30.5, and 19.5 kDa using SDS electrophoresis. Testicular tissue of the infected camels showed degeneration and necrotic changes in seminiferous tubules and interstitial tissue, along with edema and congestion. There was a significant reduction in the diameter of seminiferous tubules and germinal epithelium height. A marked reduction in testosterone levels and a high expression of HSP70 in spermatogonia, spermatocytes, Sertoli cells, and Leydig cells were observed.

**Discussion:**

Consequently, a combination of physiological and hormonal analyses may serve as a reliable indicator of *Trypanosoma* infection.

## 1 Introduction

The Arabian one-humped camel (*Camelus dromedarius* Linnaeus) is a remarkable mammal that is adapted to extreme heat and harsh desert conditions. These animals play a crucial socio-economic role, being extensively utilized across Africa and Asia for transportation, milk, wool, and meat. In Egypt, *C. dromedarius* is predominantly imported from Sudan (1). However, its reproductive inefficiency poses a significant challenge, with factors such as prolonged calving intervals, a short breeding season, delayed first service, nutritional deficiencies, pathological conditions, and low fertility rates contributing to this issue (2). Disruption in blood protein levels can adversely affect hormone secretion and gonadal function, leading to reproductive impairment (3). Therefore, regular examinations for blood parasites are essential, as severe infections can delay puberty, reduce semen quality and quantity, and reduce overall fertility.

Previous literature has identified several blood parasites in camels, including *Trypanosoma, Theileria, Babesia, Anaplasma*, and *Filaria*, all of which significantly impact productivity by reducing fertility rates and increasing mortality in heavily parasitized animals (1, 4–8). These parasites can also affect productivity indirectly by limiting food intake, weight gain, and milk production (9). Furthermore, blood parasites are recognized as common causes of infertility in male camels (6, 10, 11) and abortion in female camels (9).

Among these, trypanosomiasis, particularly caused by *Trypanosoma evansi* (Steel), is one of the most dangerous parasitic diseases affecting camels. It presents with clinical symptoms such as lethargy, loss of appetite, and poor body condition (12) and is characterized by various pathological and immunosuppressive effects (13). Recent studies have shown a strong association between trypanosomiasis, hormonal alterations, and testicular lesions (10, 11, 14). However, there is limited data regarding the impact of parasitism on serum proteins, lipid profiles, and the expression of heat shock proteins (HSPs), which are crucial for cellular protection against environmental stresses, infections, physiological disturbances, and oxidative damage (15).

HSP70, in particular, is linked to germ cell development and reproductive capacity (16), exhibiting positive expression in cellular cytoplasm, nuclei, and organelles such as mitochondria (17). Despite its importance, little is known about the relationship between HSP70 expression in the testes of *T. evansi*-infected Arabian camels and serum protein fractions.

Given these gaps in knowledge, our focus was to investigate blood protein fractions and lipid profiles in relation to hormonal disruption and testicular pathogenesis in male camels infected with *T. evansi*. Additionally, this research highlights the significance of parasitic infections as a cause of infertility by examining the expression of HSP70 in testicular tissue.

## 2 Materials and methods

### 2.1 Ethical approval

This research received ethical approval from the Faculty of Science Research Ethics Committee (FSREC) at Assiut University, Egypt, in compliance with applicable Egyptian laws regarding research and publication, under approval number (01/2023/0001). All procedures were carried out in conformity with the applicable rules and regulations. The study was conducted in accordance with the ARRIVE (Animals in Research: Reporting *In Vivo* Experiments) criteria (18).

### 2.2 Animals

A total of 107 male post-pubertal camels, aged between 5 and 10 years, were randomly selected from slaughtering house in the Bani-Adi district, Assiut Governorate, Egypt. In Assiut, camels are primarily processed at the Beni Adi slaughterhouse, with occasional slaughtering at Elhamamyah. To ensure representativeness and minimize selection bias, camels were chosen without prior knowledge of their health status or infection history. This involved selecting every nth animal presented for slaughter during the period of study. The selection of male post-pubertal camels aged 5–10 years was based on regional practices and high frequently of infection. The selected animals were examined for *Trypanosoma* infection based on clinical examinations, including general health condition, rectal temperature, and scrotal contents evaluations. Fresh blood samples were collected during the breeding season, from December 2020 to April 2021. Negative samples based on parasitological and serological examinations were used as controls with no clinical signs. It was difficult to get the owner's consent to collect a testicular Tru cut biopsy, so we had to collect samples from slaughtered animals. The testicular samples were isolated from all camels for histopathology and immunohistochemical analysis.

### 2.3 Blood collection

Five milliliters of blood were collected via jugular venipuncture using a disposable plastic syringe and a 19G needle. One portion of the blood was collected into a tube containing anticoagulant for parasitological analysis (1). The other portion was collected in tubes without anticoagulant for serum collection at room temperature until clotting occurred. Samples were transported directly on ice to the Parasitology Laboratory, Zoology Department, Faculty of Science, Assiut University, Egypt. Serum samples were collected by centrifuging the blood at 5,000 rpm for 15 min, aliquoted into dry, clean Eppendorf tubes, and stored at −80°C for further analysis.

### 2.4 Parasitological examination

Five thin blood smears were immediately prepared from each camel's blood sample, dried, and fixed with methyl alcohol. The smears were stained with diluted Giemsa and examined at high power (× 400) with oil immersion objectives using a light microscope (OPTICA, Italy). Parasite morphological identification followed the keys of Soulsby (19).

### 2.5 Serological examination

Sera samples were tested for the presence of anti-*T*. *evansi* antibodies using the card agglutination test for *T*. *evansi* (CATT/*T*. *evansi*), following the manufacturer's protocol (Institute of Tropical Medicine, Antwerp, Belgium). Approximately 45 μl of the antigen was transferred onto the test card and mixed with an equal amount of test serum diluted 1:4 with PBS (pH 7.2). The reaction mixture was agitated with a stirring rod and allowed to react on a card test rotator. Agglutination patterns were scored as negative (−), weakly positive (±), positive (+), or strongly positive (++ or +++) (20). Negative and positive controls were used for each test.

### 2.6 Quantitative analysis of serum testosterone

The concentration of serum testosterone was assessed using a commercial Enzyme-Linked Immunosorbent Assay (ELISA) kit (Human Gesellschaft für Biochemica und Diagnostica, Wiesbaden, Germany). The sensitivity and intra- and inter-assay coefficients of variation were 11% and 13%, respectively, for total testosterone concentration (21, 22).

### 2.7 Lipid profile and total protein quantification

Serum total lipids (TP), total cholesterol (TC), high-density lipoproteins (HDL-C), low-density lipoproteins (LDL-C), and triglycerides (TG) were determined using reagent tests from commercial kits (Bio Diagnostic, Egypt) and measured using a colorimetric Spectrophotometer in the Department of Zoology, Faculty of Science, Assiut University, Egypt. Total proteins were determined using a modified Bradford protein quantification assay (23). A standard curve was prepared using commercially available ovalbumin (Sigma, St. Louis, MO, USA). Each sample (100 μL) was mixed with Bradford reagent (1 mL) in a 1.5 mL microcentrifuge tube. The absorbance was measured at 595 nm using a UV spectrophotometer.

### 2.8 Proteomic analysis

Protein fractionation was evaluated by sodium dodecyl sulfate-polyacrylamide gel electrophoresis (SDS-PAGE) of *T. evansi* whole-cell antigen analysis in 12% polyacrylamide gel under reducing conditions, following the method of Laemmli (24). Briefly, 6.5 μL of each sample, 1.0 μL of reducing agent, and 2.5 μL of sample buffer were denatured in boiling water. The sample buffer contained 70% glycerol, 2% SDS, 70 mM Tris-HCl (pH 6.8), and 0.02% bromophenol blue. After gel solidification, 10 μL of the protein ladder (25–250 kDa, Sigma, Saint Louis, USA) and uninfected and infected samples were loaded into separate wells. The gel was run using an electrophoretic setup. After fractionation, the gel was stained with Coomassie brilliant blue G-250, followed by 7% acetic acid to remove excess dye, then digitized using a UV trans-illuminator (25). The size and quantities of proteins were automatically detected using the GelPro Analyser software package (version 6.3).

### 2.9 Paraffin tissue embedding

The present study was carried out on 10 representative samples of testes from infected and control animals. The testes were progressively perfused with a small amount of 10% neutral buffered formalin to prevent testicular vascular expansion. Afterwards, small blocks (1 × 1 cm) were immediately immersed in a formalin alcohol fixative for histological processing (26, 27). The fixed specimens were washed with 70% ethyl alcohol to remove remnants of the fixative and dehydrated in a graded series of ethyl alcohol [80%, 90%, 100% (I and II), 30 min each]. Dehydrated specimens were then cleared in xylene, infiltrated with paraffin, and embedded in Paraplast (Sigma Aldrich, USA). Serial 4-5 μm transverse sections were cut using a Richert Leica Microtome (RM 2125, Microsystems, Wetzlar, Germany) and incubated at 40°C to maintain dryness (28). The sections were mounted on clean glass slides, stained with Hematoxylin and Eosin, and examined using a light microscope equipped with a digital-colored camera (OPTICA 4083, Italy).

### 2.10 Morphometric analysis of testes

The average diameter of the seminiferous tubules and the height of the germinal epithelium were assessed using Image J software (29). Stained testicular sections were examined using an OPTICA light microscope and categorized by a pathologist according to the degree of degeneration. Ten fields from each slide (3 animals per group) were evaluated. Only round seminiferous tubes were randomly selected, and their diameters were measured, averaged, and expressed in micrometers. At least three measurements of the germinal height were taken in each tube, averaged, and expressed in micrometers.

### 2.11 Immunohistochemical staining of heat shock protein 70 (HSP70)

Testis from control and infected animals were subjected to immunohistochemical analysis of HSP70 according to Alnasser et al. (29). A two-step DAKO EnVisionTM+ Single reagent (Horseradish peroxidase Mouse (HRP), Agilent Technologies, Inc., Santa Clara, USA) was utilized. Section thickness of 4–5 μm sections underwent dewaxing, rehydration in descending grades of ethanol, and washing three times in phosphate buffer saline (PBS, pH 7.4) for 5 min each. A mixture of absolute methanol and drops of hydrogen peroxide (3%) was applied to sections and allowed to dry for 20 min at room temperature. The slides were washed for an additional 10 min under running water to reduce the activity of endogenous peroxidase. For antigen retrieval, the slides were immersed in a sodium citrated buffer (pH 6.0) and heated in a water bath at temperature 95–98°C for 20 min. Then, the slides were allowed to cool at room temperature for 30 min and washed three times in PBS (pH 7.4) for 5 min each. Monoclonal anti-HSP70 primary antibody (Santa Cruz Biotechnology, Cat. No. sc-32239) were applied to the slides at a dilution 1:200 for 30 min at room temperature. A ready to use Goat Anti-Mouse IgG (Abcam, Cat. No. 6789 + TM System Horseradish Peroxidase Labeled Polymer; DAKO) was applied at a dilution at 1:2,000 for 1 h at room temperature. Then, the slides were washed three times for 5 min each with PBS (pH 7.4) and treated with liquid DAB substrate chromogen system (3-amino-9-ethylcarbazole/substrate-chromogen) for 10 min at room temperature to develop a brown color at antigen sites. Sections were counterstained with Harries Hematoxylin for 30 s, dehydrated two rounds of ethanol (90% and 100%), cleared in xylene, and mounted using DPX. The immunohistochemical staining was analyzed using an OLYMPUS BX51 light microscope equipped with a digital video camera (OLYMPUS, DP72). Negative control samples were developed using an adapted standard control that excluded the use of primary antibodies.

### 2.12 CMEIAS color segmentation

CMEIAS Color Segmentation, a free, enhanced computer tool, was used to process negative photos (Supplementary material). Briefly, open image from file menu in CMEIAS Color Segmentation program, pick “Process” from the menu, and then click “Negative image” (30).

### 2.13 Statistical analysis

Data were normalized using GraphPad prism 9. The normalized data were then analyzed using SPSS version 20 (IBM, Chicago, IL, USA) and summarized by descriptive statistics for the prevalence rate. The Pearson's chi-squared test was applied to analyze the lipid profile in healthy and infected animals. One-way ANOVA followed by Duncan as *post-hoc* test, as well as independent t- test, were used to compare protein analysis and morphometric measurements between healthy and infected with 95% confidence intervals at a statistically significant level at *P* ≤ 0.05. Data were expressed as mean ± standard deviation (SD).

## 3 Results

### 3.1 Prevalence and diagnosis of *T. evansi*

**Figure 1:**
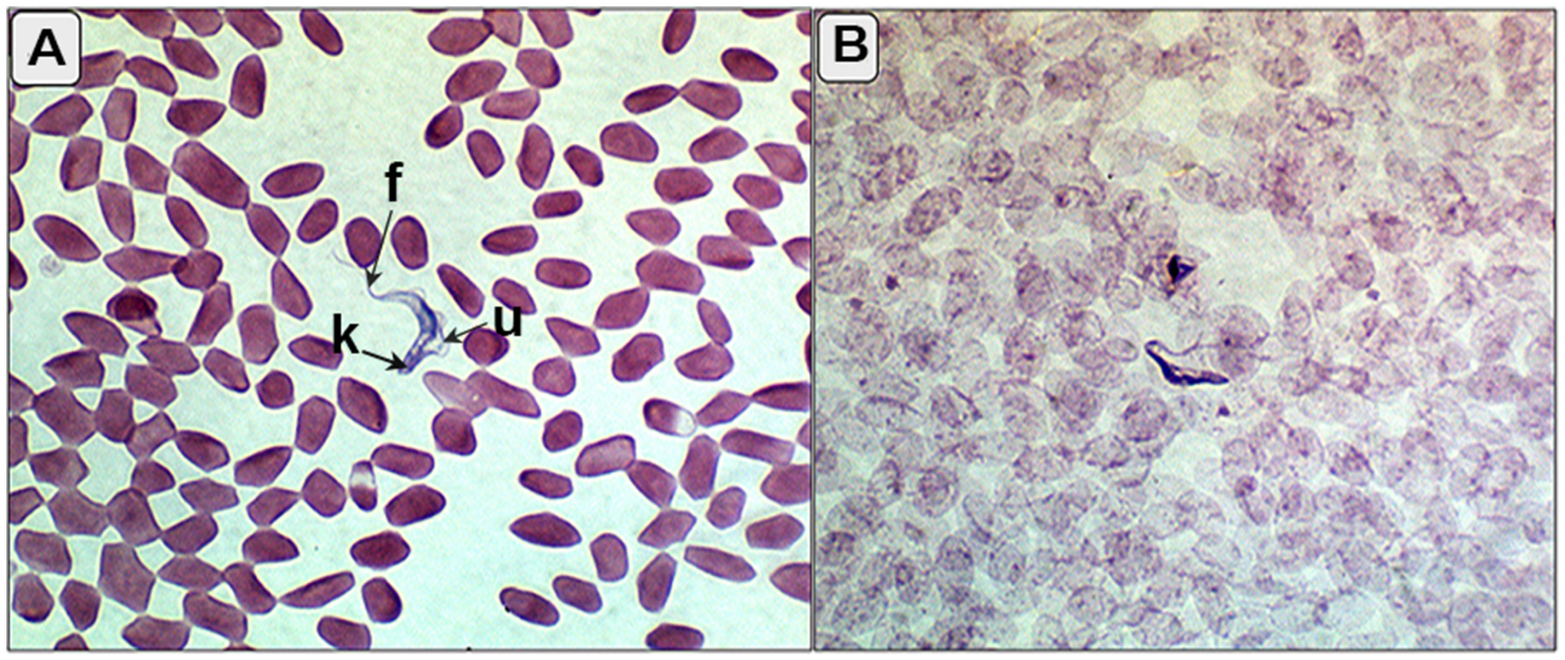
**(A, B)** Blood smear of the infected camels showing trypomastigotes (*T. evansi*) between red blood corpuscles with characterized kinetoplast (k), undulating membrane (u), and free flagella (f).

Out of 107 camels, 25 (23.4%) tested positive for *T. evansi* infection using the card agglutination test for *T. evansi* (CATT/*T. evansi*), whereas only 5 camels (4.6%) were positive in blood smear examinations (Figure 1).

### 3.2 Lipid profile

Infected camels showed a significant increase in total serum protein (*P* = 0.000). The assessment of dyslipidemia, measured as binary variables for lipid profiles (cholesterol, triglycerides, HDL, and LDL), indicated no significant increase in the risk of dyslipidemia in camels infected with *T. evansi* compared to healthy camels. The Pearson Chi-square results were as follows: cholesterol (χ^2^ = 6.0, df = 5, *P* = 0.306), triglycerides (χ^2^ = 6.0, df = 5, *P* = 0.306), HDL-cholesterol (χ^2^ = 6.0, df = 3, *P* = 0.112), and LDL-cholesterol (χ^2^ = 4.0, df = 4, *P* = 0.406).

### 3.3 SDS-PAGE proteomic analysis

**Figure 2:**
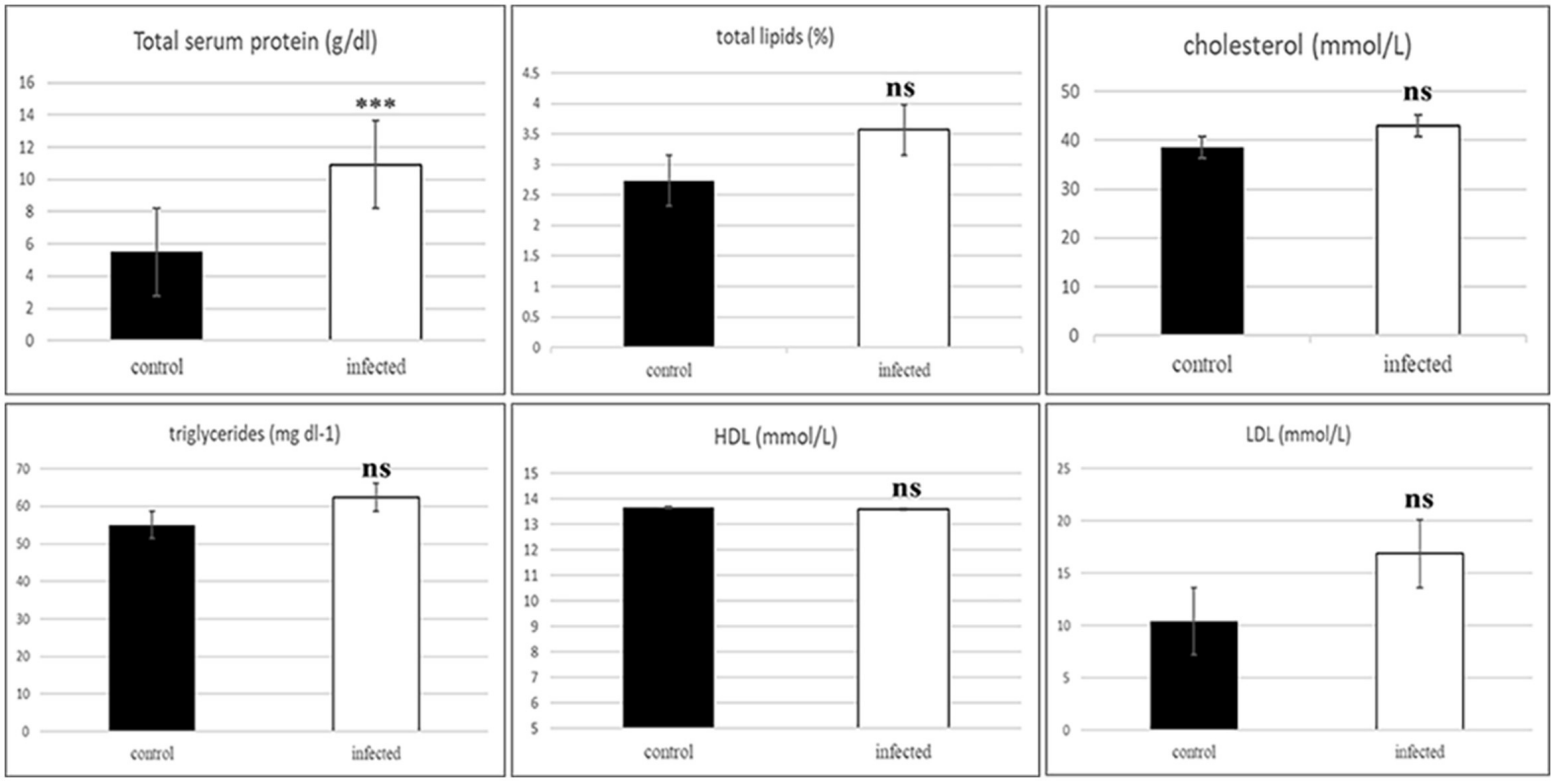
Histogram of the serum protein and binary variables for lipid profile of the healthy and infected camels showing significant increase in the total protein and non-significant in the serum lipids includes total lipids, cholesterol, triglycerides, cholesterol-HDL, and cholesterol-LDL in the infected animals. ***Indicates highly significant (*P* < 0.001), ns, indicates non-significant difference).

**Figure 3:**
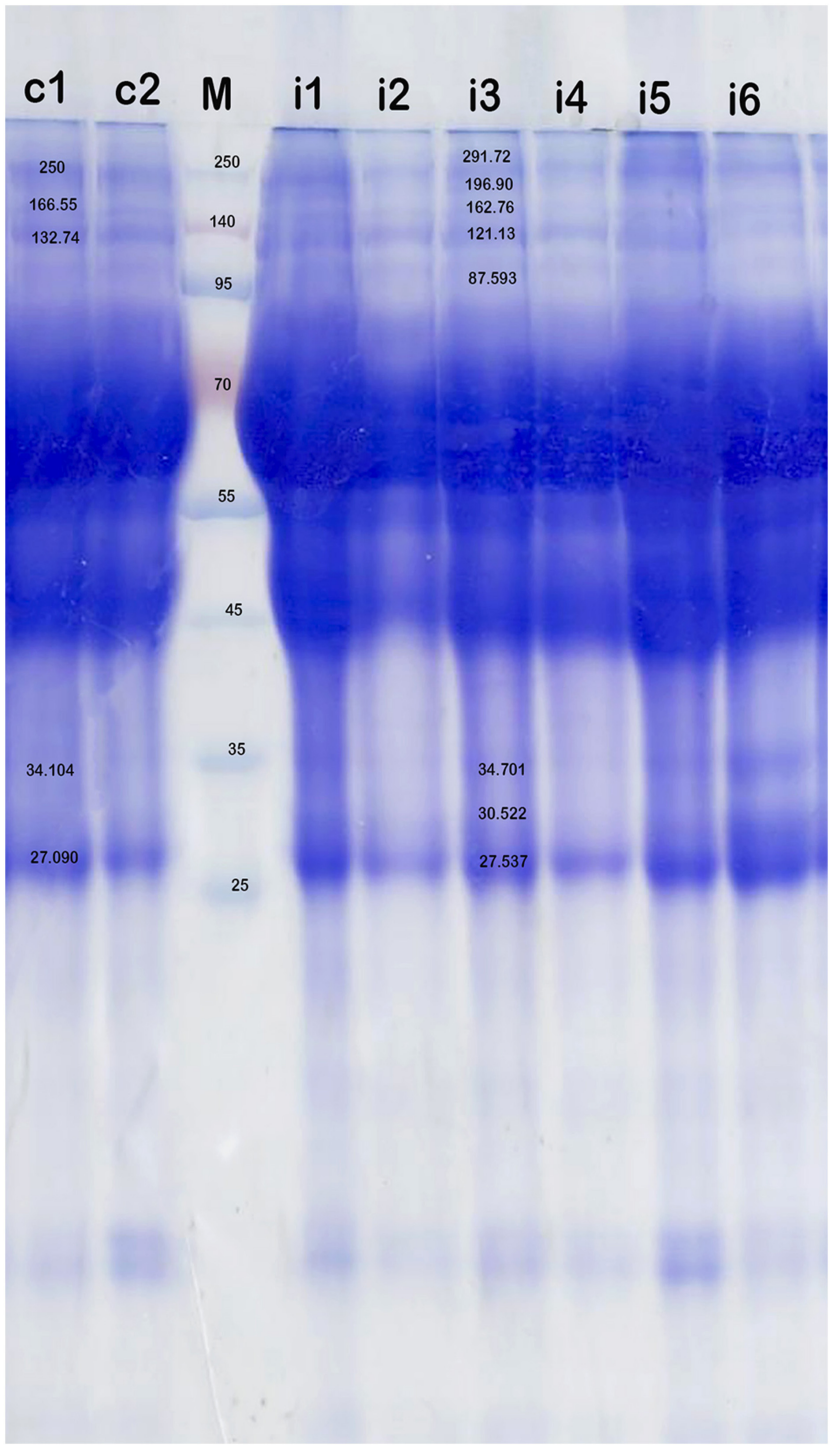
SDS-PAGE analysis of serum proteins in the health camels (c1-c2) and camels infected with *T. evansi* (i1-i6) by native gel electrophoresis (12% polyacrylamide gel) and stained with Coomassie brilliant blue. High molecular weight (>95 kDa) antigens are predominantly recognized in the infected animals. M is a protein marker.

A statistically significant increase in total serum protein levels was observed between *T. evansi*-infected and uninfected camels (t = 10.56, *P* = 0.0001, Figure 2). Electrophoretic analysis of serum proteins revealed eight major proteins bands with molecular weights (MW) ranging from 25 to 250 kDa. Of these, four major protein peaks were identified in the serum of infected camels, corresponding to MWs of 181.72 kDa (L2), 87.59 kDa (L6), 30.5 kDa (L12), and 19.5 kDa (L14), which were less prominent or absent in the serum of uninfected camels (Table 1). The high molecular weight protein (87.59 kDa) was particularly noteworthy in the serum of infected camels (Figure 3), suggesting the presence of infection-related biomarkers that may correspond to heat shock proteins (HSPs), which are known to play critical roles in thse host response to stress and infection. Conversely, a major peak at 109.5 kDa was more prominent in all serum samples of uninfected camels, representing a potential baseline protein expressed under healthy physiological conditions.

**Table 1:** Protein fractions (in percent) identified in the uninfected (control) and infected camels with *Trypanosoma evansi*.

**Lanes Groups**	**Control**			**Infected**						
**Protein fractions**	**%**									
r1	4.642637	2.05181	3.415877	5.085117	2.778363	2.888836	2.603671	2.616368	0.153649	0.829235
r2				0.962917	1.073134	1.118106	1.015828	1.562897	2.126265	1.470695
r3	1.584957	0.602417	1.591802		3.912877	2.333664	2.274419	3.74621	1.128755	
r4	3.305347	2.284116	4.331515	4.504949			2.164368			1.530945
r5	77.69924	41.37302	78.32124	2.68233	2.861337	2.073865				
r6				71.83379	73.67374	73.3485	82.07152	74.04509	76.57968	76.79526
r7										
r8										
r9										
r10	3.590892	48.24708	1.577927	3.457715	2.569757	4.682743		3.229021	5.952108	4.438909
r11										2.947883
r12				1.713205	2.863784	1.998405		2.244684	4.170101	10.00405
r13	9.175956	4.909137	7.383255	7.532176	8.715068	9.665172	8.348003	8.534352	8.461199	
r14				1.123549				1.463236		
r15			1.115924			0.43809		0.564428		1.982819
r16		0.532675	2.263094	1.104576	1.551726	1.452395	1.521712	1.993328	1.428383	

### 3.4 Serum testosterone levels

The serum testosterone concentration was significantly reduced in infected camels (2.675 ± 1.47 ng/mL) compared to uninfected camels (4.763 ± 0.65 ng/mL), indicating hormonal disruption.

### 3.5 Morphopathological lesions of testes

**Figure 4:**
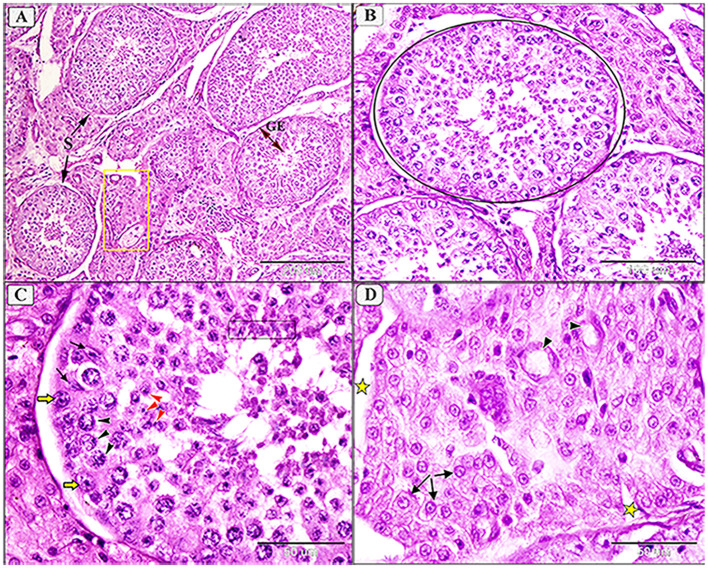
Photomicrograph showing normal histological architecture of testis in camel, *C. dromedarius*, showing: **(A)** Normal seminiferous tubules (S) full of spermatogenic cells (GE) and healthy interstitial tissue containing Lyding cells (yellow square); **(B)** High magnification of identical round seminiferous tubules with normal size; **(C)** Higher magnification showing different stages of spermatogenesis, spermatogonia (yellow arrow), primary spermatocytes (black arrowhead), secondary spermatocytes (red arrowhead), spermatids (black oblong), and many spermatocytes in the lumen; **(D)** Higher magnification of the interstitial tissue with Lyding cells (arrows) and dilated blood vessels (star).

Grossly, most examined testes appeared normal, though some displayed congestion. Microscopically, normal camel testes comprise numerous seminiferous tubules enclosed by a basal lamina and myoid cells (Figures 4A, B). Each seminiferous tubule is lined with germinal epithelium containing Sertoli cells and various spermatogenic cells at all stages of development, including spermatogonia, spermatocytes, and spermatids, with abundant spermatocytes in the lumen (Figure 4C). Interstitial spaces contain numerous oval Leydig cells surrounding the seminiferous tubule (Figure 4D).

**Figure 5:**
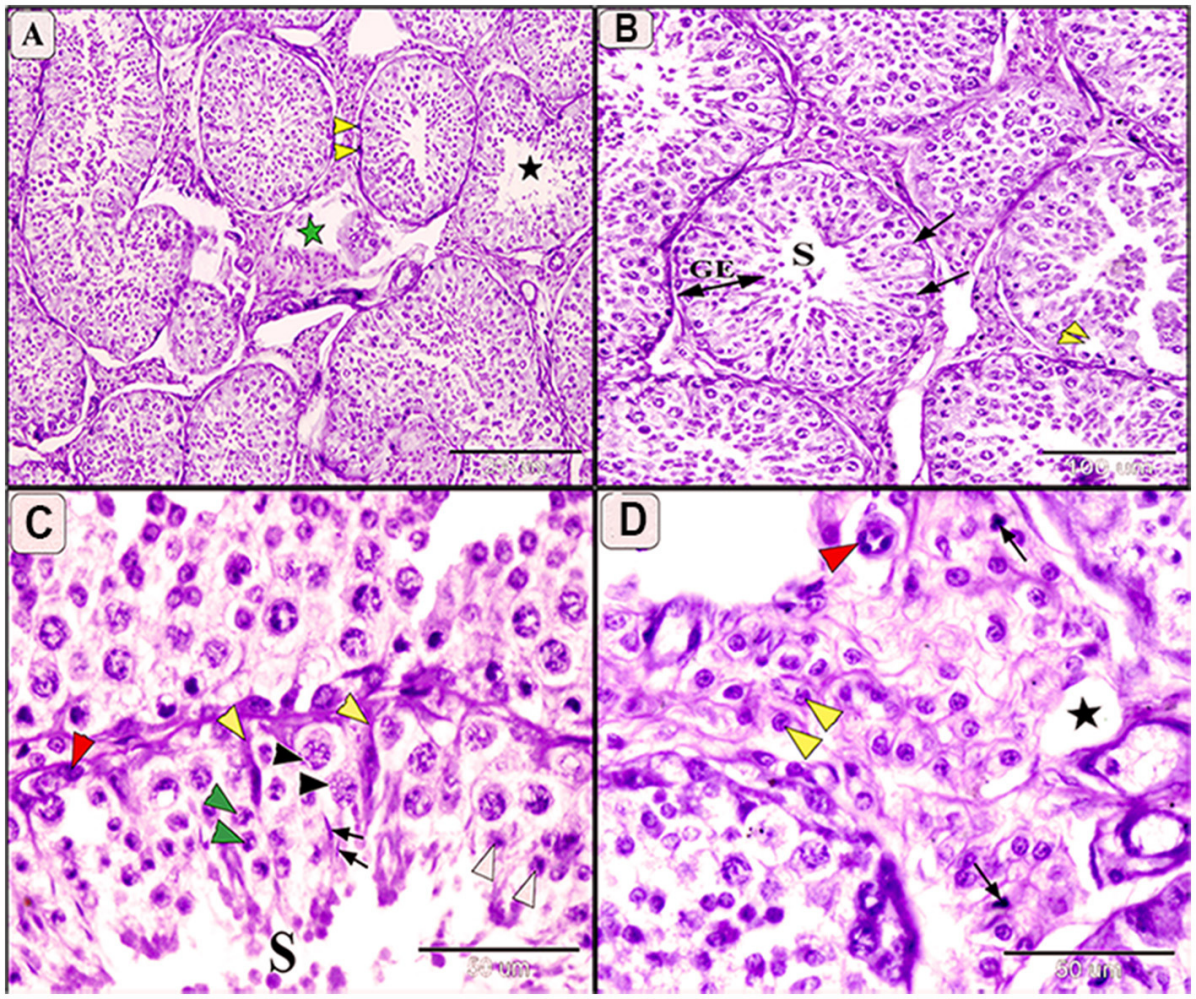
Photomicrograph of testis in camels infected with *T. evansi* showing mild degeneration in the testis of camel, *Camelus dromedarius*, showing: **(A)** Convoluted some of the seminiferous tubules, thickening the basement membrane (yellow arrowhead), inactive spermatogenesis process (black star), and necrobiotic changes in the interstitial tissue (green star); **(B)** Higher magnification showing seminiferous tubule (S), decreased the height of spermatogenic cells (GE), and mild degeneration of some Sertoli cells (arrow); **(C)** Higher magnification showing mild degeneration in spermatogenic cell, spermatogonia (red arrowhead), primary spermatocytes (black arrowhead), secondary spermatocytes (white arrowhead), spermatids (arrow), and increase size of Sertoli cells with enlarged nuclei and light eosinophilic cytoplasm (yellow arrowhead); **(D)** Higher magnification of the interstitial tissue showing decrease in the size of Lyding cells (yellow arrowhead), pyknosis of some cell (arrow), edema (star), and plumbing of the endothelial cells (red arrowhead).

**Figure 6:**
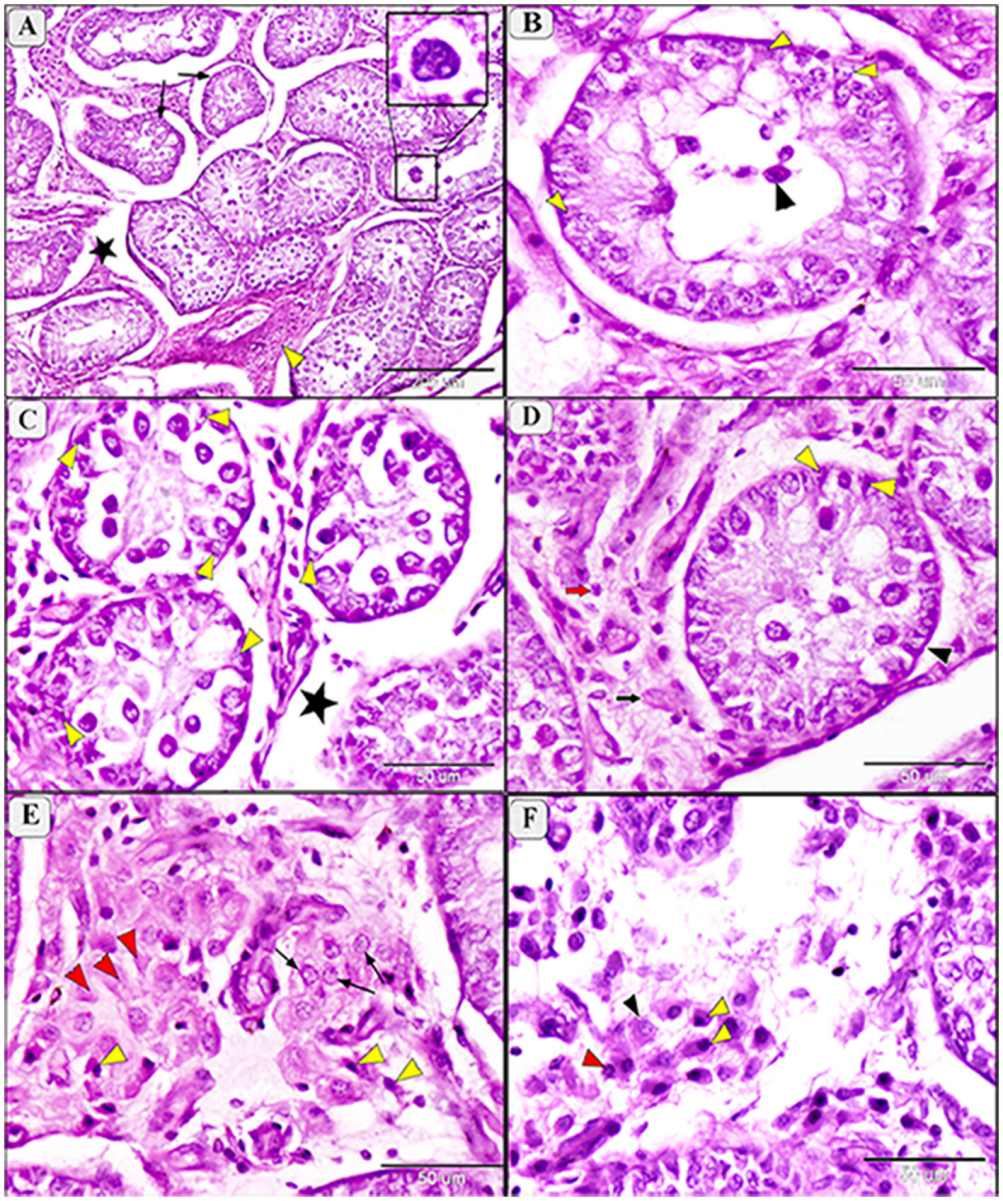
Photomicrograph of testis in camels infected with *T. evansi* showing moderate degeneration in the testis of camel, *Camelus dromedarius*, showing: **(A)** Highly convoluted and degenerative seminiferous tubules (arrow), edema (star) and necrobiotic changes (yellow arrowhead) of the interstitial tissue with formation of giant cells (higher magnification in the square); **(B)** Higher magnification showing a high loss of germinal cells and Sertoli cells (yellow arrowhead) and incomplete spermatogenesis (black arrowhead); **(C)** Necrobiotic changes in germinal epithelium (yellow arrowhead) and edema of the interstitial tissue (star); **(D)** Depletion of spermatogenic epithelium, increase thickening of the basement membrane (black arrowhead), degenerative Sertoli cells (yellow arrowhead), necrobiosis (black arrow) and pyknosis (red arrow) of Lyding cells; **(E)** Higher magnification of the interstitial tissue showing necrobiotic changes (red arrowhead), pyknosis (yellow arrowhead), and vacuolar degeneration (arrow) of Lyding cells; **(F)** Higher magnification showing edema in the interstitial tissue with vacuolar degeneration (black arrowhead) and pyknosis (yellow arrowhead) of Lyding cells.

In infected camels, histological changes ranged from mild to moderate degeneration of seminiferous tubules and germinal epithelium (Figures 5, 6). Mild degeneration was characterized by disorganized and convoluted seminiferous tubules (Figure 5A) and inactive spermatogenesis (Figures 5A–C). The interstitial edema of endothelial cell were observed, indicating fluid accumulation due to inflammatory reaction. Moreover, pyknosis of Leydig cells, characterized by condensation and fragmentation of the cell nucleus, often indication cell death (Figure 5D).

Moderate degeneration involved significant loss of germinal cells and incomplete spermatogenesis (Figures 6A, B) and was associated with depletion of the spermatogenic epithelium, desquamation of spermatogenic cells, and necrosis in the germinal epithelium (Figure 6C). Sertoli cells appeared enlarger, with enlarged nuclei and light eosinophilic cytoplasm, often accompanied by azoospermia (Figure 6C). Additional features of moderate degeneration include interstitial edema with mononuclear inflammatory cell infiltration (Figures 6D, E), multinucleated giant cell formation (Figure 6A), prominent peritubular edema, necrotic degeneration, and nuclear pyknosis (Figure 6F).

**Figure 7:**
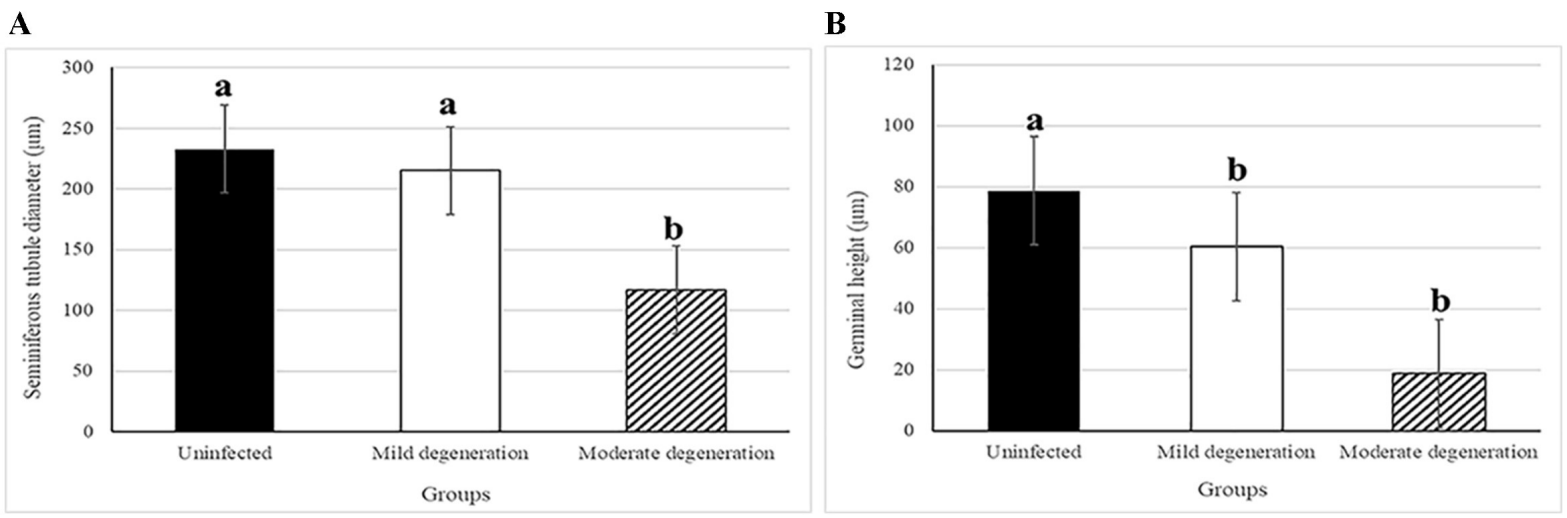
Histogram showing morphometric analysis of testicular degeneration in camels, *C. dromedarius*, infected with *T. evansi* showing: **(A)** Statistically significant reduction in the diameter of seminiferous tubules in moderate degeneration; **(B)** Statistically significant reduction in the germinal height in the mild and moderate degeneration degree compared to uninfected animals. Data are expressed as micrometer (μm), different letter refer to significant difference between groups.

Morphometric analysis revealed a non-significant reduction in the diameter of seminiferous tubules during mild degeneration (ANOVA, *P* = 0.139), while a significant reduction (ANOVA, *F* = 56.15, *P* < 0.0001) was observed in moderate degeneration (Figure 7). Both mild and moderate degeneration were associated with a highly significant decrease (ANOVA, F = 76.01, *P* < 0.0001) in germinal heights (Figure 7).

### 3.6 Expression of anti HSP70 in testes of infected camels

**Figure 8:**
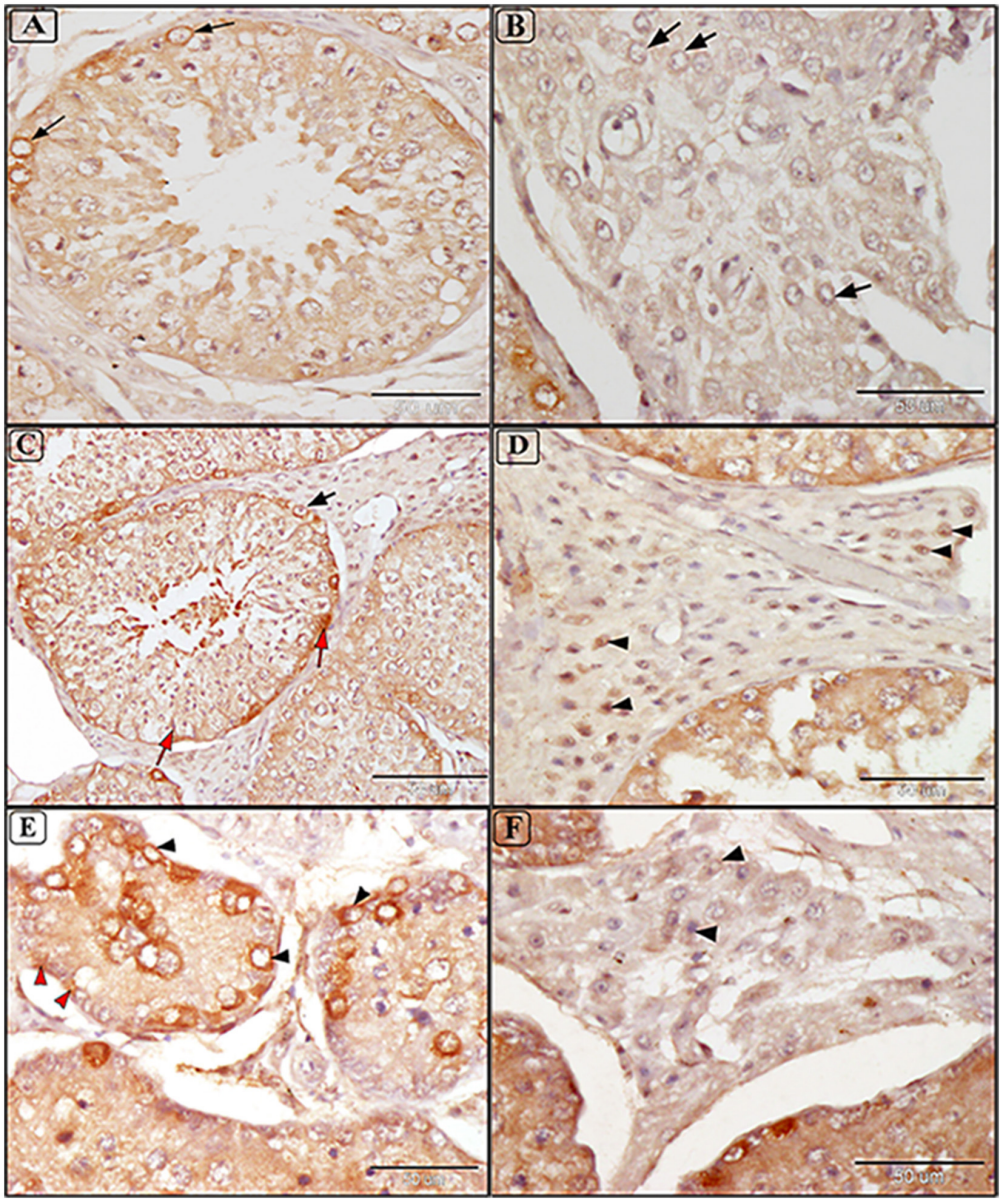
Immunostaining expression of HSP70 in the testis of camels infected with *T. evansi* showing: **(A, B)** Faint positive expression of anti-HSP70 are shown in cytoplasmic brown color in spermatogonia Lyding cells in the interstitial tissue (arrow) of normal uninfected camels (a small box represents the negative control); **(C, D)** Mild testicular degeneration in camels, *C. dromedarius*, infected with *T. evansi* showing moderate positive expression of anti-HSP70 in different germ cells including spermatogonia (black arrow), spermatids, spermatozoa, and Sertoli cells (red arrow); **(C)** and Lyding cells in the interstitial tissue (arrowhead) **(D)**; **(E, F)** Moderate testicular degeneration in camels, *C. dromedarius*, infected with *T. evansi* showing severe positive expression of anti-HSP70 in all cells and interstitial tissue (arrowhead).

Tissue expression of HSP70 was positive in all examined samples, with immunoreactivity indicated by a brown color localized mainly in the membrane and cytoplasm of cells. Mild and focal positive expressions were observed in spermatogonia and interstitial tissue in the testes of some uninfected camels (Figures 8A, B). Infected camels exhibited increased HSP70 expression, particularly in spermatogenic cells and Sertoli cells in mildly degenerative testes (Figure 8C). Leydig cells in the interstitial tissue showed faint membranous HSP70 immunoreactivity (Figure 8D). In moderately degenerative testes, strong immunostaining for HSP70 was observed in spermatogonia, spermatocytes, Sertoli cells, and Leydig cells (Figures 8E, F).

## 4 Discussion

*Trypanosoma evansi* is a pathogen that silently cripples Egypt's livestock industry. Trypanosomiasis, a zoonotic disease, is often correlated with bacterial infections transmitted by biting flies and hard ticks (31). In the present study, the prevalence of *T*. *evansi* infection in the examined camels using Giemsa smear examination 4.65%, which lower than those recently reported in Egypt (8.3%) (32), Saudi Arabia (33), Sudan (34), Kenya (35), Algeria (36), and Nigeria (37). Using CATT technique, 23.4% of examined camels were positive, suggesting high specificity and sensitivity of molecular analysis. These findings align with the previous records in different regions in Egypt (5, 8, 12, 13, 31), though high incidence of non-clinical infection was found (31%) of *T. evansi* in Upper Egypt (38). On the other hand, our findings were higher rate than those reported in Egypt (8), Somalia (39), and Iran (40), as well as lower than those reported in Egypt (41), Saudi Arabia (42), Iran (43), and Kenya (35). Variations in detection rates could be attributed to climate conditions, vector viability and activity, differing methodologies (31), and chronic nature of *T. evansi* infection in Egypt (44). Sobhy et al. (45) demonstrated that camels on the northwest coast may infected with *T. evansi* from hard ticks and *Stomoxys* flies. Moreover, significant amounts of *T. evansi* were reported in the skin of experimentally infected mice without detectable parasitemia (46), suggesting that the skin of negative animals might serve as a silent source of infection. The high prevalence of *T. evansi* highlights the need for regular screening and control camel challenges, which can lead to significant productivity and economic losses. We acknowledge that this prevalence rate represents a substantial proportion of infected camels, indicating that *T. evansi* is a prevalent health concern in the region. This rate suggests that a notable number of camels are at risk of the associated health issues, including infertility and other reproductive impairments. In Egypt, high prevalence rates of trypanosomes infection were detected during summer and spring, with increase vector density influenced by high temperature (12, 45). Moreover, adult camels (< 4 years old) were more frequently infected with *T. evansi* than young camels (47), which align with the present study. Thus, camels within this age range are more likely to exhibit the physiological, biochemical, and pathological effects associated with chronic *T. evansi* infections.

The main finding of the current study is the close correlation between parasitism, serum protein concentrations, dyslipidemia, and testicular degeneration, alongside the upregulation of HSP70 expression. These interrelated factors present significant challenges to camel reproduction and productivity. Specifically, parasitism load and its associated physiological disruptions such as serum protein imbalances and alteration of lipid metabolism, which can lead to testicular degeneration, ultimately affecting fertility. This aligns with previous studies, which have highlighted the impact correlation between high parasitism and other diseases, including pleuropneumonia, mange, tuberculosis (TB), and trypanosomiasis, on camel fertility and overall productivity (48). Lipids, beyond their energy-storing role, are crucial for cell function, sperm maturation, and hormone production (49). Hamad et al. (50) reported that no significant effect of season on triglycerides and cholesterol. Moreover, little variations were reported between rut and non-rut seasons in healthy camels (51). Our results showed a non-significant increase in total serum lipids, cholesterol, triglycerides, and LDL/HDL ratio in the infected camels compared to the healthy animals, which still hint at underlying biological processes that warrant further investigation. Moreover, an imbalance of fatty acids, including cholesterol, indicate shifts in metabolic states that may affect the overall reproductive health such as dyslipidemia, systemic oxidative stress, poor quality gametes, and infertility (52). Ferrieres (53) demonstrated that dyslipidemia, including hypertriglyceridemia, hypercholesterolemia, and reduced HDL-cholesterol, is correlated to various pathophysiological conditions. Conversely, El-Bahr and El-Deeb (54) reported significant decrease in triacylglycerol, cholesterol, HDL/cholesterol with significant increase in LDL/cholesterol in camels naturally acute infected with *T. evansi*. This variation could be due to the chronicity of infection and age of the examined camels. Although limited data is provided about the effect of trypanosomiasis and serum lipid profile, the imbalance of serum lipids in the current study may indicate sperm dysfunction under parasitism. The correlation between lipid profile and reproductive hormones, as highlighted by Bobjer et al. (55), suggested that there may be functional implications between serum triglycerides and testosterone levels in men with azoospermia. Additionally, Jones et al. (56) reported that sperm sensitivity to lipid peroxidation, due to high polyunsaturated fatty acids. This is linked to sperm mutation, dysfunction, and subsequently fertility disorders.

The breeding season is characterized by significant hormonal fluctuations, which may affect the immune system and potentially modulate susceptibility of animals to infections. Our results indicate that healthy camels aged 5 to 10 years have a high testosterone concentration (4.763 ± 0.65), whereas *T. evansi*-infected camels significantly show reduction in the concentration of serum testosterone (2.675 ± 1.47). Consistent with previous findings, our study revealed hypercholesterolemia and hypertriglyceridemia are associated with reduced sperm count, increased abnormalities, decreased testosterone levels, and testes weight (57). Similar hormonal changes were reported in *T. evansi*-infected camels, with significant reduction in luteinizing hormone and follicle-stimulating hormone, and increased cortisol level (10). Mohammed et al. (14) demonstrated similar effects in *T. evansi* infected rats and human infected with *T. gambiense* (58) which linked to hypercortisolemia impairing gonadotropin-releasing hormone secretion. These physiological changes could influence the prevalence or detectability of the infection, thus potentially impacting the representativeness of the present results.

Hematological profiles and chemical compositions have been documented in camels infected with various blood parasites, including *Theileria* (59), *Babesia* (60), and *Typanosoma* (61). However, the mechanism by which parasitism affects serum proteins is not well-studied. In our study, comparative SDS-PAGE analysis showed four major peaks in the serum of infected animals with molecular weights 181.72, 87.59, 30.5, and 19.5 kDa. The small molecular weight proteins 30.5 kDa and 19.5 kDa may be represent acute-phase or stress-related proteins such as small heat shock proteins. These findings align with Hoter et al. (62) who reported small molecular weight, ranging from 12 to 43 kDa in the Arabian camel (*C. dromedarius*). Additionally, the serum of *T. evansi*-infected camels exhibited a protein band at 87.59 kDa, which possibly correspond to high HSP expression. This is concurred with Rudramurthy et al. (63) who revealed the immunoreactivity of the expressed protein (~70 kDa) in serum of animals infected with *T. evansi*. On the other hand, Ulmasov et al. (64) demonstrated strong induction of a 73kDa protein coupled with HSP88 and HSP 60 after heat stress on camel lymphocytes. These findings highlight significant alterations in serum proteins patterns associated with *T. evansi* infection, with high molecular weight proteins and specific peaks serving as potential biomarkers of infection.

Amin et al. (10) indicated that immune responses influence sex hormones and contribute to infertility typically concurrent with trypanosomiasis. About 90% of blood testosterone is produced from testes (10). Our findings revealed that testes of *T. evansi*-infected camels exhibit different levels of degenerative changes in seminiferous tubules. *T. evansi*-infected deer showed the same degeneration in the seminiferous tubules and sperms (65). Sekoni (66) reported that such pathological changes reduce the reproductive capacity in males, causing infertility with chronicity of the parasitism. The experimental rats infected with *T. evansi* exhibited increase in oxidative stress and lipid peroxidation in the testes, suggesting the cell injury and confirmed the pathogenicity of this parasites (11). These pathological lesions in the testicular tissue might be attributed to decrease levels of circulating luteinizing hormone and follicle-stimulating hormone due to the hypothalamic-pituitary-gonadal axis is unable to self-regulate (11). Moreover, the infected animals showed high temperature which id directly associated with parasitemia (67, 68). Since the testicular tissue is highly sensitive to temperature, the hyperthermia may have contributed to the testicular lesions such as necrosis, supporting our findings that *T. evansi* can reduce infertility either directly or indirectly.

A marked reduction in the number of seminiferous tubules with incomplete spermatogenesis and necrosis of lining epithelium were also recorded. Besides, a marked increase in the size and number of Sertoli and Lyding cells was observed. Additionally, interstitial tissue showed edema with infiltration of inflammatory cells, necrosis, and pyknosis of Lyding cells. In this aspect, the pathological changes in Lyding cells and interstitial tissue were significantly correlated with the testosterone concentration. Such results have coincided with the previous report by Amin et al. (10). Additionally, our results show a significant reduction in the diameter of seminiferous tubules and germinal height in the mild and moderate testicular degeneration of infected animals, which aligned with Bataineh and Nusier (69). This significant decrease may be attributed to the reduction of serum testosterone level due to decrease luteinizing hormone. Friedländer et al. (70) reported that testosterone production is most likely impaired only in the final stage of the Leydig cell's development during the non-breeding season. This difference is attributed to Lyding cells necrobiosis and interstitial tissue edema.

Heat shock proteins (HSPs) are essential for thermotolerance and maintaining protein homeostasis (62). The HSP70 famile, which includes both inducible (HSP72) and constitutive (HSP73) forms, plays a key role in cellular stress responses (71). Our study utilized goat anti-mouse secondary antibodies, closely related to camel HSPs. According to the phylogenetic analysis of mammalian HSPs, camel HSPs have close related to ruminant HSPs including goats (72). Cytoplasmic localization of HSP70 in spermatocytes and spermatids was noted in both normal and infected animals, aligning with Feng et al. (73) findings of HSP70 expression in normal and maturation-arrest testis. Immunohistochemical results indicate that increased HSP70 in the different germ cells (spermatogonia, spermatocytes, Sertoli cells, and Lyding cells) correlates with the degree of degeneration. This can be attributed to increase thermal stress *T. evansi* prevalence and their side effects.

Previous studies indicated that HSP 72 kDa and 73 kDa were structurally similar, having key role in the survival of cell, as well as stress severity and recovery process (74). Parallel to our SDS-PAGE results, we reported distinctive expression of HSP70 in the testes of *T. evansi*-infected camels, which can be used as cellular injury biomarker (75). Microscopic examination in our study revealed significant HSP70 cytoplasmic localization in spermatogonia, Sertoli cells, spermatocytes, and Lyding cells, which were tightly associated with cellular pathogenesis. Overexpression of HSPs reflects cellular stress responses, apoptosis, and cell survival, impacting animal performance and productivity (76). Moreover, HSPs could be considered as main diagnostic marker to trypanosomiasis due to increase of body temperature linked to parasitism peak (67). Our results align with Dix et al. (77) who reported that HSP70 gene upregulation linked to meiosis failure, germ cell apoptosis, and male infertility in mice. Additionally, upregulation of HSP70 has been linked to progress and development of inflammatory diseases including atherosclerosis (78). This could be attributed to the most of HSP genes do not have introns (79), which allow for quick expression and explain how they can be produced when RNA splicing is disrupted by stressors (80). Moreover, HSP70 have multiple cytoprotective functions including folding new proteins, refolding of denatured proteins, prevent aggregation of denatured proteins, translocation of proteins across membranous organelles, and anti-apoptotic factors (81).

Acute and chronic camel trypanosomiasis could cause anemia, loss weight, anoxia, weakness, increase body temperature up to 41°C, and immunosuppression, and perhaps death if left untreated (2, 67, 82). Moreover, it causes economic impacts including decreased production (a 30% decrease in milk and meat yield morbidity), high tendency of abortions in all age groups, and a 3% death rate (83–85).

## 5 Conclusion

This study explored the correlation between physiological analyses, parasitism, and pathological lesions in the testes of camels. Key findings included patterns of altered serum protein levels, dyslipidemia, HSP70 expression as a diagnostic marker to trypanosomiasis. However, the study's findings should be interpreted considering potential limitation, such as sample size, variability in parasitic load, and reliance on random sampling from a slaughterhouse, which may not represent the overall camel population. While the study provides valuable insights into the effects of *T. evansi* infection, the findings may not fully represent the physiological baseline outside the breeding season. Further research employing advanced proteomic techniques are necessary to further identify and characterize the serum protein of *T. evansi*- infected camels, enabling a deeper understanding of their roles in infection and immune response.

## Data Availability

The datasets presented in this study can be found in online repositories. The names of the repository/repositories and accession number(s) can be found in the article/Supplementary material.
