# Supplementary material for: Impact of trypanosomiasis on male camel infertility
Source: Front Vet Sci. 2025 Jan 16;11:1506532. doi: 10.3389/fvets.2024.1506532 (PMC11780594; doi:10.3389/fvets.2024.1506532)
Supplement: Supplementary file 1 [file Data_Sheet_1.pdf]

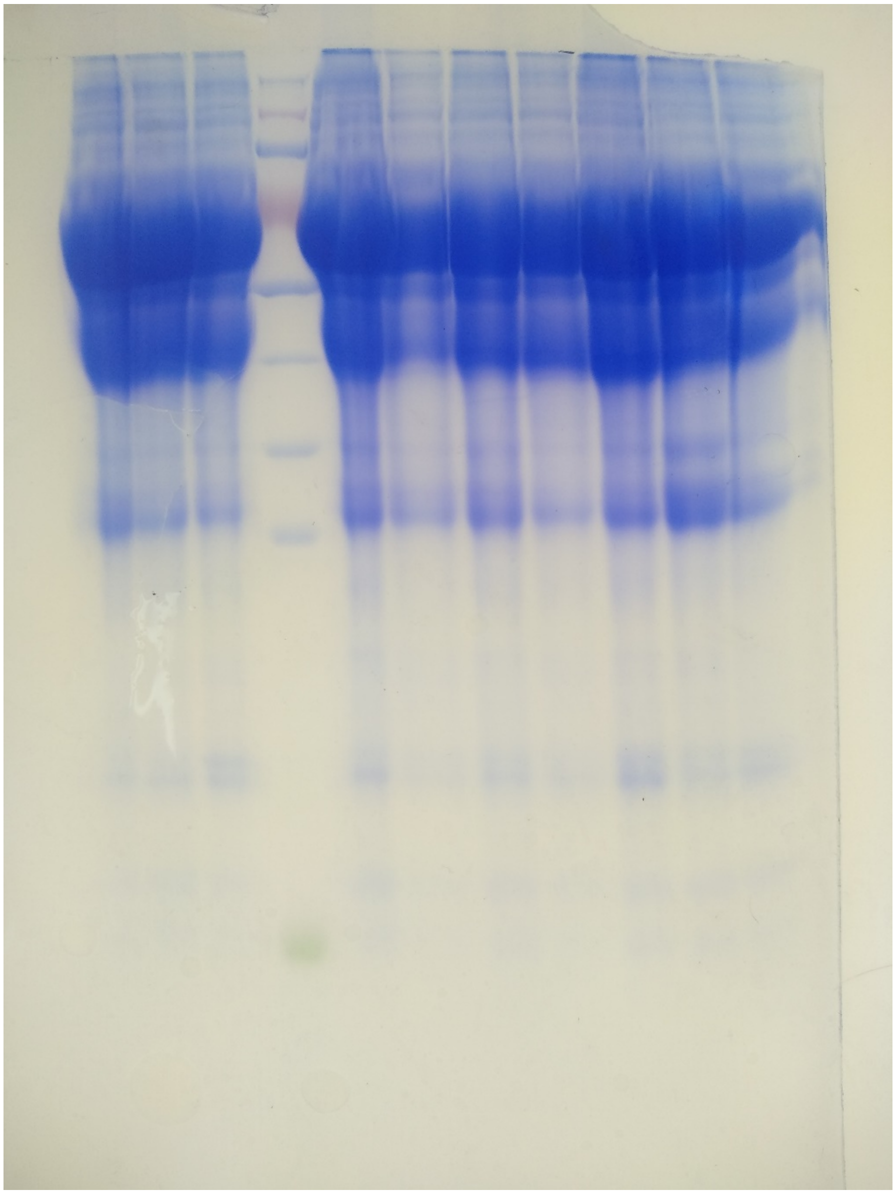

Figure S1: the original electrophoresis gel

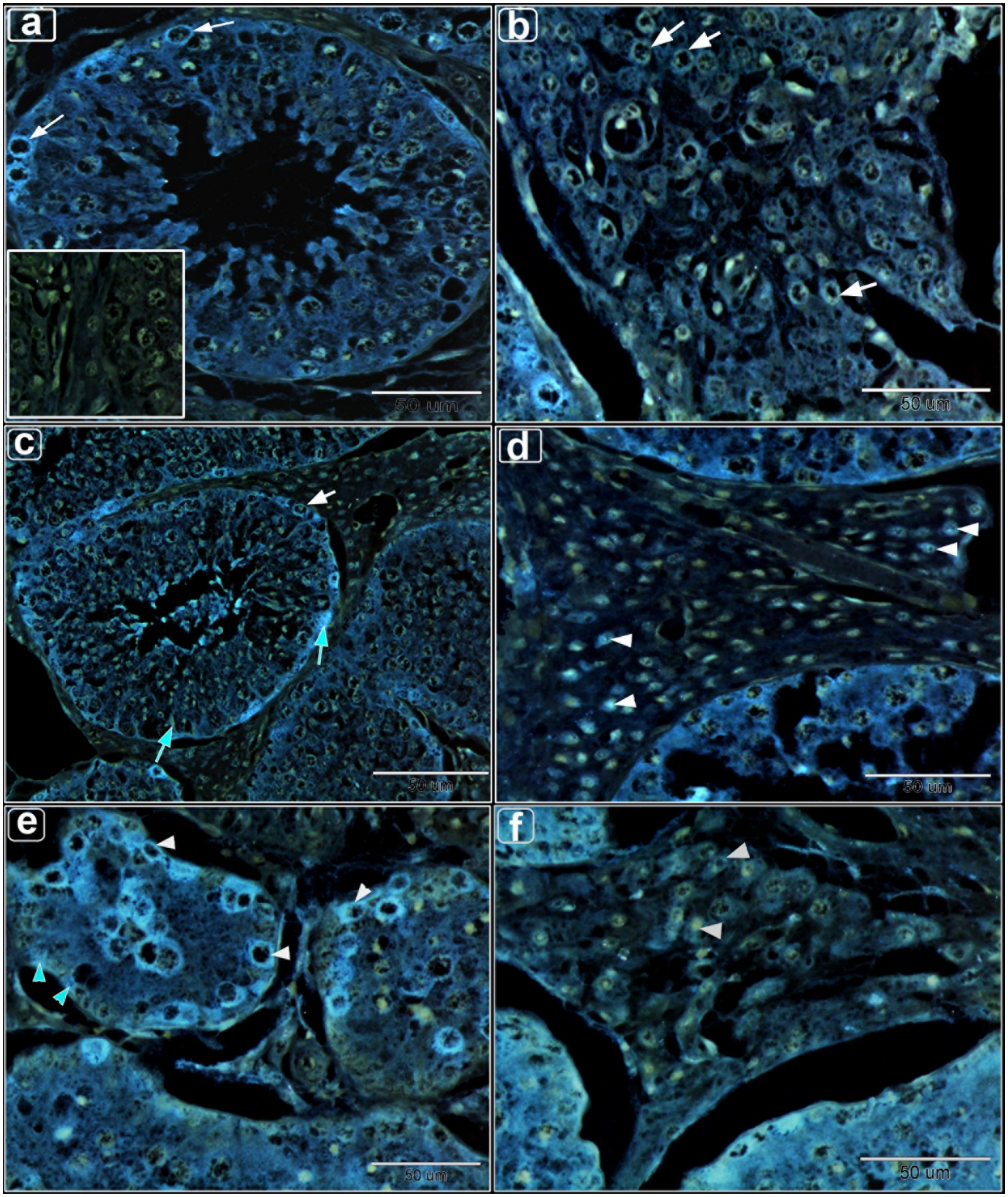

Figure S2: Negative picture of immunostaining of HSP70 using CMEIAS Color Segmentation showing positive expression.
